# Automation — down to the nuts and bolts

**DOI:** 10.1155/S1463924600000201

**Published:** 2000

**Authors:** Robert J. Fix Sr, Jannell M. Rowe, Bain C. McConnell

**Affiliations:** R. J . Reynolds Tobacco Co. Winston-Salem NC 27102-1487 USA

## Abstract

Laboratories that once viewed automation as an expensive luxury are now looking to automation as a solution to increase sample throughput, to help ensure data integrity and to improve laboratory safety. The question is no longer, ‘Should we automate?’, but ‘How should we approach automation?’ A laboratory may choose from three approaches when deciding to automate: (1) contract with a third party vendor to produce a turnkey system, (2) develop and fabricate the system in-house or (3) some combination of approaches (1) and (2). The best approach for a given laboratory depends upon its available resources. The first lesson to be learned in automation is that no matter how straightforward an idea appears in the beginning, the solution will not be realized until many complex problems have been resolved. Issues dealing with sample vessel manipulation, liquid handling and system control must be addressed before a final design can be developed. This requires expertise in engineering, electronics, programming and chemistry. Therefore, the team concept of automation should be employed to help ensure success. This presentation discusses the advantages and disadvantages of the three approaches to automation. The development of an automated sample handling and control system for the STAR™ System focused microwave will be used to illustrate the complexities encountered in a seemingly simple project, and to highlight the importance of the team concept to automation no matter which approach is taken. The STAR™ System focused microwave from CEM Corporation is an open vessel digestion system with six microwave cells. This system is used to prepare samples for trace metal determination. The automated sample handling was developed around a XYZ motorized gantry system. Grippers were specially designed to perform several different functions and to provide feedback to the control software. Software was written in Visual Basic 5.0 to control the movement of the samples and the operation and monitoring of the STAR™ microwave. This software also provides a continuous update of the system's status to the computer screen. The system provides unattended preparation of up to 59 samples per run.


Journal of Automated Methods & Management in Chemistry, Vol. 22, No. 5 (September–October 2000) pp. 133–138

### Automation — down to the nuts and bolts

**Robert J. Fix Sr, Jannell M. Rowe, and Bain C. McConnell**

*R. J. Reynolds Tobacco Co., Winston-Salem, NC 27102-1487, USA*

*Laboratories that once viewed automation as an expensive luxury are now looking to automation as a solution to increase sample throughput, to help ensure data integrity and to improve laboratory safety. The question is no longer, 'Should we automate?', but 'How should we approach automation?' A laboratory may choose from three approaches when deciding to automate: (1) contract with a third party vendor to produce a turnkey system, (2) develop and fabricate the system in-house or (3) some combination of approaches (1) and (2). The best approach for a given laboratory depends upon its available resources. The first lesson to be learned in automation is that no matter how straightforward an idea appears in the beginning, the solution will not be realized until many complex problems have been resolved. Issues dealing with sample vessel manipulation, liquid handling and system control must be addressed before a final design can be developed. This requires expertise in engineering, electronics, programming and chemistry. Therefore, the team concept of automation should be employed to help ensure success. This presentation discusses the advantages and disadvantages of the three approaches to automation. The development of an automated sample handling and control system for the STAR™ System focused microwave will be used to illustrate the complexities encountered in a seemingly simple project, and to highlight the importance of the team concept to automation no matter which approach is taken. The STAR™ System focused microwave from CEM Corporation is an open vessel digestion system with six microwave cells. This system is used to prepare samples for trace metal determination. The automated sample handling was developed around a XYZ motorized gantry system. Grippers were specially designed to perform several different functions and to provide feedback to the control software. Software was written in Visual Basic 5.0 to control the movement of the samples and the operation and monitoring of the STAR™ microwave. This software also provides a continuous update of the system's status to the computer screen. The system provides unattended preparation of up to 59 samples per run.*

### Introduction

For the corporate environment, the 1990s has been a decade of downsizing. Cutting labour costs is viewed as one way to better position a company to compete in today's global marketplace. This strategy, along with increased regulation, has placed greater pressures on analytical and quality assurance laboratories to produce more data, at a faster rate and with fewer human resources. Laboratories that only a few years ago viewed automation as an expensive luxury are today seriously looking to automation as one solution to increase sample throughput, to help ensure data integrity and to improve laboratory safety. The question no longer is 'Should we automate?' but 'How should we approach automation?'

In the mid-1980s, our analytical laboratories began to look at robotics and automation as viable tools to help to reduce the repetitive tasks required in preparing samples for analysis. One individual was assigned to automation. His first task was to develop a plan. As he viewed automation, there were three paths to choose from: (1) a contract with a third party vendor to produce turnkey systems, (2) design and fabricate systems in-house or (3) some combination of approaches (1) and (2). Each has its advantages and disadvantages. We chose path (3).

After the first two projects proved to be less than successful, it became evident that we needed to rethink our automation effort. Although we did not experience the success we had hoped for, we did learn a valuable lesson that helped to ensure a more favourable outcome for succeeding projects. No matter how straightforward an idea appears in the beginning, the solution will not be realized until many complex problems have been resolved. There was not an individual in the analytical department with the expertise to be a mechanical designer, electronics engineer, computer programmer, chemist and 'dreamer'. Therefore, an interdepartmental team was assembled. The team is comprised of one person from each of the above mentioned disciplines plus a representative from the financial department.

Since the formation of our Automation Team, several large and small projects have been completed successfully. The latest project, an automated sample handling and control system for the STAR™ System focused microwave, was transferred to our Elemental Analysis laboratory in February 2000. This project is a good illustration of how a seemingly simple 'pick and place' procedure can quickly become very complicated once the requirements are understood.

### Choosing an approach to automation

Throughout the 1970s, in an attempt to keep pace with sample demands, we began to look for ways to increase productivity. In many of our laboratories sample preparation consumed much of the analyst's workday. Eliminating some or all of the manual steps involved in sample preparation would result in a significant increase in productivity. However, the lack of available technology made it impractical to attempt to automate most of the manual procedures. Then, in the spring of 1982, Zymark introduced the first Zymate Laboratory Automation System [1]. This marked the beginning of a new era in laboratory automation. The introduction of robotics into the laboratory meant that it was now feasible to automate some of the routine, time-consuming tasks involved in sample preparation.

We looked at three approaches to automation and compared the advantages and disadvantages of each:

1. *Contract with a third party vendor for a turn-key system*

Advantages:

- we would not have to tie up our personnel designing and building a system;
- we would have the benefit of expertise that we lacked.

Disadvantages:

- we would have less control over the final system;
- it would be more difficult to make mid-course corrections;
- we would be dependent on the vendor to maintain and/or modify the system.

2. *Design and build the system in-house*

Advantages:

- we would have total control over the final system;
- we could modify the system, if needed, during the development;
- we would be able to maintain and/or modify the system, as needs dictate.

Disadvantages:

- we would have to tie up a great deal of our facility's resources;
- we might spend time and resources developing automation that others have already developed;
- time would be needed to develop new skills.

3. *A combination of* 1 *and* 2

Advantages:

- we would have many of the advantages of the first two approaches by building on the success of others as we develop our skills.

Disadvantages:

- we would experience some of the disadvantages of both approaches; however, these would be minimized by levering our knowledge with outside vendors.

Within our R&D department, there exists a mechanical design division, an electronics division and a state-of-the-art machine shop. Therefore, we decided that the third approach offered the most advantages. During the planning stage of a project, we would determine what equipment should be purchased, then design and fabricate everything else. For example, if a project required a robot, it would be a waste of time and effort to develop our own. Instead, we would purchase the robot and then develop workstations to go along with it.

### *The team concept*

Within all organizations, there exist invisible barriers between departments. Each department tends to have a different set of priorities. It soon became evident that a single individual from the analytical division could not effectively circumvent these barriers. Trying to coordinate work from different departments, without any real authority to do so, was next to impossible. Senior management had to be convinced that devoting company resources to laboratory automation would produce positive benefits. The solution was to form an interdepartmental team. In 1993, management accepted a proposal to form an R&D Laboratory Automation Team. The team consists of six permanent members, representing each of the disciplines and departments that are critical to completing a project (figure 1). At the start of each project, one member of the team is designated as project manager. During a project other personnel are added to the team as needed. The analyst performing the procedure being automated becomes the team's 'Client' and serves as an equal member of the team until the project is completed.

**Co-Chairs**

Automation/Robotic Expert — Electronic Design Engineer

**Remaining Team Members**

Chemist

Mechanical Design Engineer

Computer Programmer

Financial Analyst

Client

*Figure 1. R&D Laboratory Automation Team.*

Three basic rules were established to guide the team. First, management determines priorities. Second, financial justification (a cost/benefit analysis) shall be completed before any substantial monies are allocated. Third, the team must be in full agreement of a design before presenting it to management.

Since its formation, the Automation Team has successfully implemented several small and large systems for different divisions in R&D. These systems have resulted in increased safety, greater sample throughput and improved data integrity. The latest system to be implemented, an automated sample handling and control system for the STAR™ focused microwave, demonstrated the importance of the team concept in developing automated solutions.

### The STAR™ Automated System

Sample preparation is the most time-consuming and labour intensive step in the process of elemental analysis. Microwave digestion, using concentrated acid to prepare samples for analysis, has done much to alleviate this problem. Open-vessel microwave systems reduced the time required for sample decomposition. However, the analyst had to be present during the 2 to 4 hour procedure to add reagents to the samples and to make sure that they did not go to dryness.

In 1995, our Elemental Analysis laboratory became a test site for CEM Corporation's STAR™ System focused microwave (figure 2). The STAR™ is an open-vessel digestion system with 6 microwave cells, capable of operating independently using individual methods and temperature feedback control. It has automated reagent addition and vapour containment. The STAR™ reduced our digestion time to 20 minutes per sample. Although

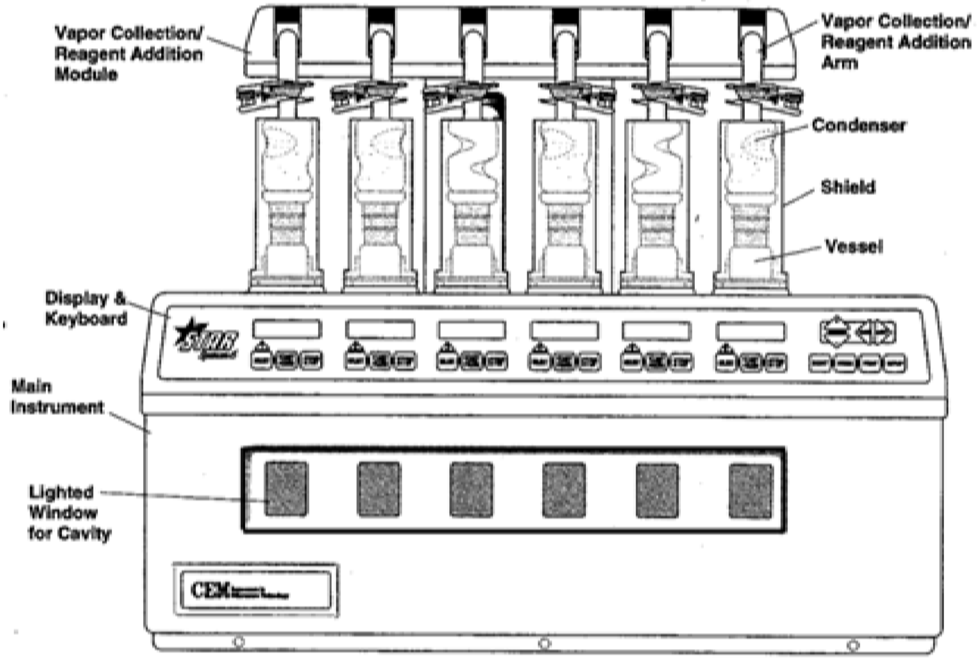

*Figure 2. The STAR™ System.*

this instrument reduced the time and manual steps required for sample preparation, the analyst still had to be present to feed samples to the system.

The automation team was asked to determine the feasibility of automating the sample loading. At first glance, it appeared to be a simple, straightforward pick and place procedure. A 250 ml Pyrex tube with a glass condenser would be picked up from a rack and placed into one of the microwave cells. An exhaust arm would be lowered onto the condenser. After digestion, the exhaust arm would be raised, the tube and condenser removed from the microwave and placed back into the tube rack. The team agreed to take on the project.

The system requirements were determined during the conceptual design phase. During this phase, the 'Client' requested more options for the system and raised some safety concerns. A number of issues dealing with mechanical manipulation of the glassware, monitoring the STAR™ system and safety had to be resolved. These issues were addressed by dividing the project into four individual tasks: (1) material handling, (2) mechanical design of the work envelope, (3) gripper design, and (4) software development for sequencing of events and system control. A different team member was assigned to head up each task. As problems arose, team members met to resolve them.

### Material handling

The material handling requirements consisted of manipulating three different objects to fixed points in the work envelope. None of the motions was complex and they required only 4 degrees of freedom. Although a surplus seven-axis cartesian robot was available, the team decided to purchase a new three-axis linear actuator from Intelligent Actuator of America.

The benefits of the linear actuator outweighed the initial capital investment. For example, the system came pre-configured for our application and required simple assembly to be operational. The actuators were mounted above the table surface, minimizing the overall footprint and maximizing the usable work envelope. The positioning repeatability of all three axes was specified at 0.003 inches. This resolution was accurate enough for all positioning tasks.

The system is made up of three standard length actuators (one 1800 mm and two 500 mm) connected together. The 1800mm actuator axis is ~36 inches above the table surface and defines the longer 'X' envelope (table width). One 500mm actuator is mounted to the 'X' actuator and serves to move the payload in the 'Y' direction (table depth). The other 500 mm actuator provides a 'Z' axis perpendicular to the table surface. The 'Z' axis is also equipped with an electronic brake that prevents the payload from falling during absence of power.

Each actuator is driven by an AC servo motor with built-in incremental encoder. All motors are connected to a single three-axis controller. Digital inputs and outputs on the controller were also utilized for all discrete control. Programming for motion control and input/output actuation is split between the motor controller and the PC. All movement end positions are stored in a point table in the motor controller. Movement instructions consist of a pre-determined set of commands communicated through the controller's RS-232 serial port. Each instruction received is decoded into one of the following command types: home all axes, point to point linear move, point to

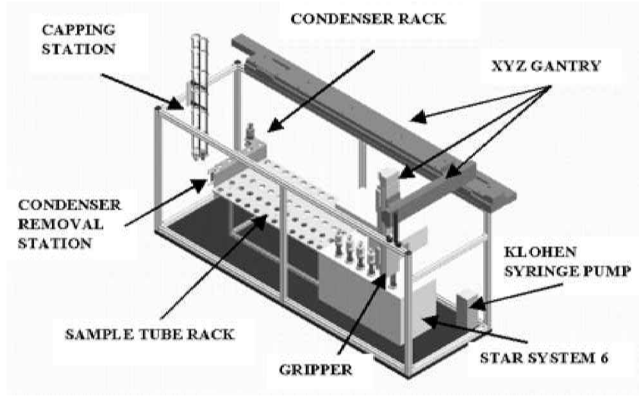

*Figure 3. Diagram of the automated STAR™* System 6.

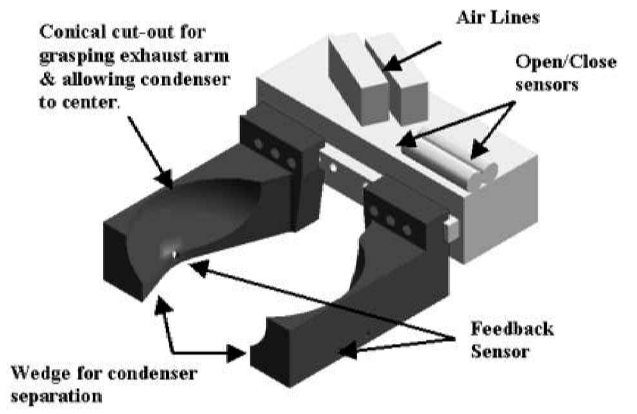

*Figure 4. Multifunctional gripper.*

point to point arc move, turn output bit on, turn output bit off, read state of input bit, read controller status.

The control program on the PC issues motion commands. A status check command is used to determine the completion of a previous motion command. Bit commands are issued from the PC for gripper opening and closing, vessel detection, vacuum pump operation and to turn off power to the oven if an excessive temperature is detected.

*Mechanical design and work envelope*

The system layout is shown in figure 3. To the left of the STAR™ microwave is a 60-position sample tube rack. The rack holds 59 samples for digestion. The last position on the rack holds a wash tube that is used to flush the reagent lines of the STAR™ system with distilled water after completion of the run. The rack is made from Teflon to eliminate damage from contact with acid. Attached on the left of the sample tube rack is a six-position rack for storing condensers. At first we considered having a condenser on each sample tube. This would have made programming simpler, but would have prevented capping the sample tubes after digestion. Testing verified that a condenser could be reused without cleaning. Therefore, we decided to transfer the condenser from each completed sample tube to the next sample tube to go into the microwave. This necessitated the need for a condenser removal station. This station is located on the front end of the condenser rack.

The capping station, positioned on the far left, consists of two racks of caps. The caps are made from Teflon and are designed to fit into the top of the sample tubes.

A Kloehn 50300 syringe pump (Kloehn Company Ltd, Las Vegas, NV) with an RS232 port and an eight-port valve is in the back right corner. Tubes run from the pump into each of the exhaust arms on the STAR™. After digestion of a sample is complete, and before the tube is removed from the microwave, distilled deionized water is added to the sample tube by the Kloehn pump. The amount of dilution water is entered by the analyst at the start of each run. Internal standards are added to each sample by the analyst prior to analysis of the samples.

A panel box (not shown) is attached to the right of the system. This box contains the motor controllers and power supply for the gantry as well as the power supply for the Kloehn pump.

*The gripper*

During the preliminary design stage, the gripper was considered to be one of the simplest parts of the system. However, it became the single most important part of the system. The final design of the system required the gripper to perform five functions. These are: (1) move the condensers and place them on to the sample tubes, (2) move the sample tubes to and from the microwave, (3) raise and lower the exhaust arms of the STAR™ unit, (4) remove the condensers from the sample tubes after the digestion and cap the completed sample, and (5) provide feedback to the control software.

A Robohand parallel gripper, equipped with a sensor to indicate open and closed states, was purchased from Piedmont Technical Sales, Charlotte, NC. The fingers were designed to allow the gripper to perform all five tasks (figure 4). Two features of the gripper allow it to perform four of its tasks: (1) each finger has a conical shaped cutout and (2) the bottom of the fingers is designed as a wedge. The gripper picks up a condenser by closing around the condenser's top, which is also conical in shape. The fingers do not close tight enough to apply any force to the condenser. This allows the condenser to be centred before being lowered into the sample tube. The wedge portion closes tightly around the sample tube. If the tube is not perfectly straight when being lowered into the microwave or when being moved back into the sample rack, the conical surface area that is in contact with the tube will cause it to straighten as it is lowered, instead of binding. The conical shaped portion of the fingers enables the gripper to close around specially designed handles attached to the exhaust arms. Since the exhaust arms move in an arc, the handle is allowed to slide in the fingers as it moves up or down. After sample digestion, the sample tube and condenser are moved to a separation station. The gripper is positioned just above the top of the tube. As the fingers close, the wedge portion of the gripper forces the condenser up as the tube is held into place. A Teflon sleeve on the

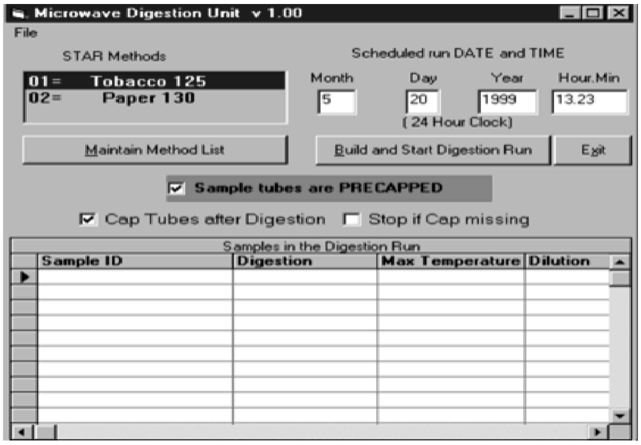

*Figure 5. Run Setup screen*

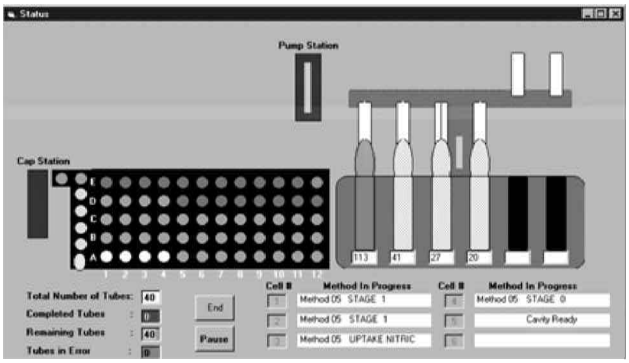

*Figure 6. Run Status Screen showing a run that has just been started. Acid is being added to the tube in the third cell of the STAR™. Two of the microwave cells are still empty and exhaust arms are shown in the raised position.*

ground glass joint of the condenser aids in the separation by preventing ‘freezing’ of the joint. The gripper opens and moves to pick up the loosened condenser by its top and places it on to another sample tube or back into the condenser rack.

In the bottom centre of the fingers is a Keyence Fiber Photoelectric sensor connected to an amplifier unit. Detection is in ‘through beam’ mode with multiple outputs. The feedback that this provides allows the control program to know what was picked up (i.e. sample tube, sample tube with a condenser or sample tube with a cap). Once the gripper has moved to a drop off location, the control program can determine that the object picked up is still present. If the object is not present at its destination, the system will enter a shutdown sequence. This is a critical safety feature of the system.

### *Software*

The control software is written in Visual Basic 5.0 (Microsoft Corp., Richmond, WA). It consists of two main forms: (1) Run Setup and (2) System Status and Control.

The Run Setup form allows the analyst to build a run, which consists of sample identifications (IDs), the STAR™ method or methods to be used for the digestion, the maximum temperature the sample can reach and how much to dilute the sample (figure 5). Each sample ID may be digested using a different STAR™ method. (The STAR™ is capable of storing up to 20 methods.) The analyst has the option of starting the run immediately or on a time delay. If the analyst wants to run samples without IDs then he clicks on ‘File’. A menu is displayed that gives the option of ‘Blank Run’. If this option is selected, then only one STAR™ method can be selected. The list of STAR™ methods is maintained from this screen. Clicking on the ‘Build and Start Digestion Run’ transfers the run setup to the system control program and displays the System Status and Control screen.

The System Status and Control screen displays the events as they take place (figure 6). At the start of a run, the control program queries the first microwave cell to make sure that the exhaust arm is in the up position. If it is, then the first available condenser is placed on to the first sample tube. The tube is transferred to the cell, the exhaust arm is lowered and the cell’s digestion program started. This is repeated until all six cells are filled. The control program monitors each cell, and displays the temperature and the stage of the method. As the temperature increases for a given sample, the colour on the display gradually changes to red. If the temperature of a cell exceeds the maximum temperature allowed, the control program turns off the cell, records the failure and eliminates that cell from further use. If more than two samples overheat, the system enters a shutdown mode. This allows the remaining cells to complete their operation before being turned off.

When a sample reaches completion, it remains in the cell until a cool-down temperature is reached (104 °C). The Kloehn syringe pump adds the deionized water for dilution. During this step the water is pumped through a tube inside the exhaust arm which rinses down the sides of the condenser. The tube and condenser are removed from the cell and transferred to the condenser removal station. The condenser is removed and placed on the next sample tube. If there are no more samples, the condenser is placed into the condenser rack. The completed sample is removed from the condenser removal station, capped and placed back in its correct position in the tube rack. After the last sample is completed, a run file is stored on the hard drive. This file contains the information about each sample: which microwave cell it was digested in, the highest temperature reached and any errors that may have occurred during processing.

### Summary

The automated system for focused microwave digestion of samples has been successfully implemented in the Elemental Analysis laboratory (figure 7). It runs unattended and can digest up to 59 samples in 7 hours, freeing the analyst to direct attention to other areas of work in the laboratory. Although the project appeared relatively simple at first, it proved to be quite complicated. This project demonstrated the importance of a team effort in developing automation. Without the combined efforts of

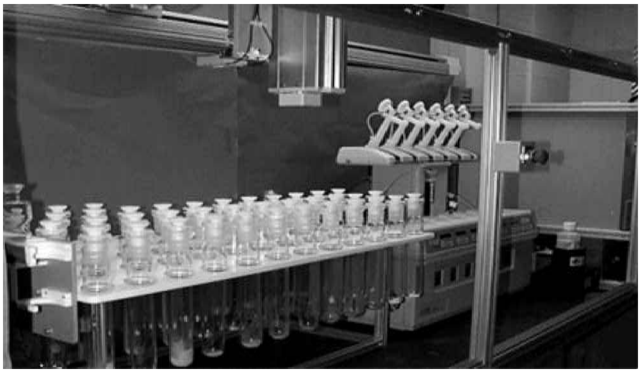

*Figure 7. Automated system for focused microwave digestion of samples*

people with expertise in several disciplines, this system would still be in the development stage.

Our automation efforts had a tenuous beginning. Once a team was assembled that brought together all of the different departments needed to complete a project, we began to realize success. Since 1993, the team has completed 13 projects. These projects have resulted in increased safety, annual savings of around $200 000 and annual labour savings of 3 manpower-years.

The most valuable lesson the team has learned is, 'When it comes to automation, nothing is as simple as it may seem in the beginning'. Therefore, proper and thorough planning is the most essential step in any automation project.

### Acknowledgements

We wish to acknowledge the contributions of our co-workers on the automation team whose efforts made the successful completion of this project possible. They are: Mark DeBusk, Jack Nelson, John Thompson and Nancy Huettle.

### Trademarks

STAR, CEM Corp., Matthews, NC, Teflon, Du Pont Corp. Visual Basic, Microsoft

### Reference

1. Zenie, F. H., 1984, Trends in automation – technology and economics. *Advances In Laboratory Automation – Robotics.*

